# Spectral clustering identifies patterns of chiropractic care in a national longitudinal cohort

**DOI:** 10.1093/jamiaopen/ooag035

**Published:** 2026-05-14

**Authors:** Monika Ray, Shao-You Fang, Anthony J Lisi, Patrick S Romano

**Affiliations:** Department of Internal Medicine, University of California Davis, CA, Sacramento, California, 95817, United States; Center for Healthcare Policy and Research, University of California Davis, CA, Sacramento, California, 95817, United States; Center for Healthcare Policy and Research, University of California Davis, CA, Sacramento, California, 95817, United States; Department of Biomedical Informatics and Data Science, Yale School of Medicine, New Haven, Connecticut, 06520, United States; Department of Internal Medicine, University of California Davis, CA, Sacramento, California, 95817, United States; Center for Healthcare Policy and Research, University of California Davis, CA, Sacramento, California, 95817, United States

**Keywords:** Neck pain, low back pain, spectral clustering, explainable boosting machines, generalised additive model, hierarchical group lasso regularisation, feature selection, patient clusters, chiropractic care, administrative claims data

## Abstract

**Objective:**

Characterise longitudinal patterns of chiropractic visits for neck pain or low back pain by using machine learning (ML) methods and explainable models.

**Data and Methods:**

Using de-identified claims data from 2016 to 2023 for adults from the Optum Labs Data Warehouse, we applied spectral clustering (SC) to identify novel patient clusters. Then we used explainable boosting machines (EBM) for feature ranking followed by hierarchical group lasso regression for feature selection. A logistic regression model used for parameter estimates.

**Results:**

SC identified 3 clusters—low, moderate and high dose—based on their pattern of chiropractic visits. An interesting finding was a small cluster where patients received persistently higher care for several months. Age, gender and number of prior visits to a chiropractor, primary care provider, or physical therapist emerged as strong indicators for provider type and frequency of visits.

**Discussion:**

Patients receiving spinal manipulative therapy sorted into 3 markedly different trajectories of utilisation. This unexpected variation mandates further investigation to identify optimal dose based on patient and provider characteristics. We also present EBM, a robust alternative to computationally heavy feature selection methods, to identify features necessary for predictive models. This approach obviates the need for opaque feature selection methods.

**Conclusion:**

Results show the use of advanced, explainable methods to discover knowledge that can be missed by other methods. We present an approach to identify hidden patterns in large data that can guide hypothesis driven research. Our work can identify factors that drive high utilisation of services and inform practice guidelines.

## Background and significance

Low back pain (LBP) and neck pain (NP) conditions collectively impose an extremely large burden on the healthcare system. In the United States, spinal conditions rank among the top 3 drivers of national healthcare spending, with high prevalence, substantial disability, and lost productivity. Current clinical guidelines recommend a number of non-pharmacologic treatments as key front-line therapies, most of which are commonly delivered by chiropractors.^1^ A study by Kazis et al. reported that in a cohort of new LBP treatment episodes among 216 504 commercial insurance and Medicare Advantage enrollees, 23% of the cases were initiated with chiropractors, second only to primary care providers at 53%^2^ while a study by Fenton et al. reported that among 770 326 new NP treatment episodes, chiropractors were the most common initial provider (45%), followed by primary care providers (33%).^3^ Despite these large studies, there are many unanswered questions regarding the timing and frequency of chiropractic visits.^4–6^ In this work, we sought to characterise distinct chiropractic visits after an index chiropractic visit for LBP, NP or combined NP/LBP diagnosis in a large US cohort spanning 7 years (2016-2023). We use machine learning methods to discover patterns in large datasets and then use explainable, robust, systematic feature selection methods to identify features for healthcare models.

The advantages of using unsupervised machine learning (ML) methods, such as spectral clustering (SC), on extremely large datasets, is that they do not impose distribution assumptions.^7–9^ The goal of unsupervised ML is knowledge discovery which leads to revealing novel patterns that get missed in hypothesis driven research.^10^ Clustering, which groups similar datapoints, helps highlight the characteristics of data^11^ while hierarchical group lasso is a state-of-the-art method for feature selection,^12^^,^^13^ which is useful for creating parsimonious models. However, it is computationally demanding and time consuming on large datasets. Highly accurate models such as deep neural networks, support vector machines, etc. come at the cost of high complexity and non-interpretability. Meanwhile, methods depending solely on *P*-values are flawed and models using backward/forward stepwise regression are prone to model instability and bias.^14–19^ Therefore, we show the use of an explainable feature ranking method which is robust, yet lightweight, the Explainable Boosting Machine (EBM).^20^ EBMs are efficient for exploratory analyses and have the properties desirable in healthcare, and public policy research, such as transparency, explainability, etc.

## Objective

Our goal was—(1) Use unsupervised ML to identify distinct chiropractic care patterns, and then (2) use a “glassbox” method, which is a generalised additive model, to identify features—with the condition that each phase would remain interpretable.

## Materials and methods

**Figure 1:**
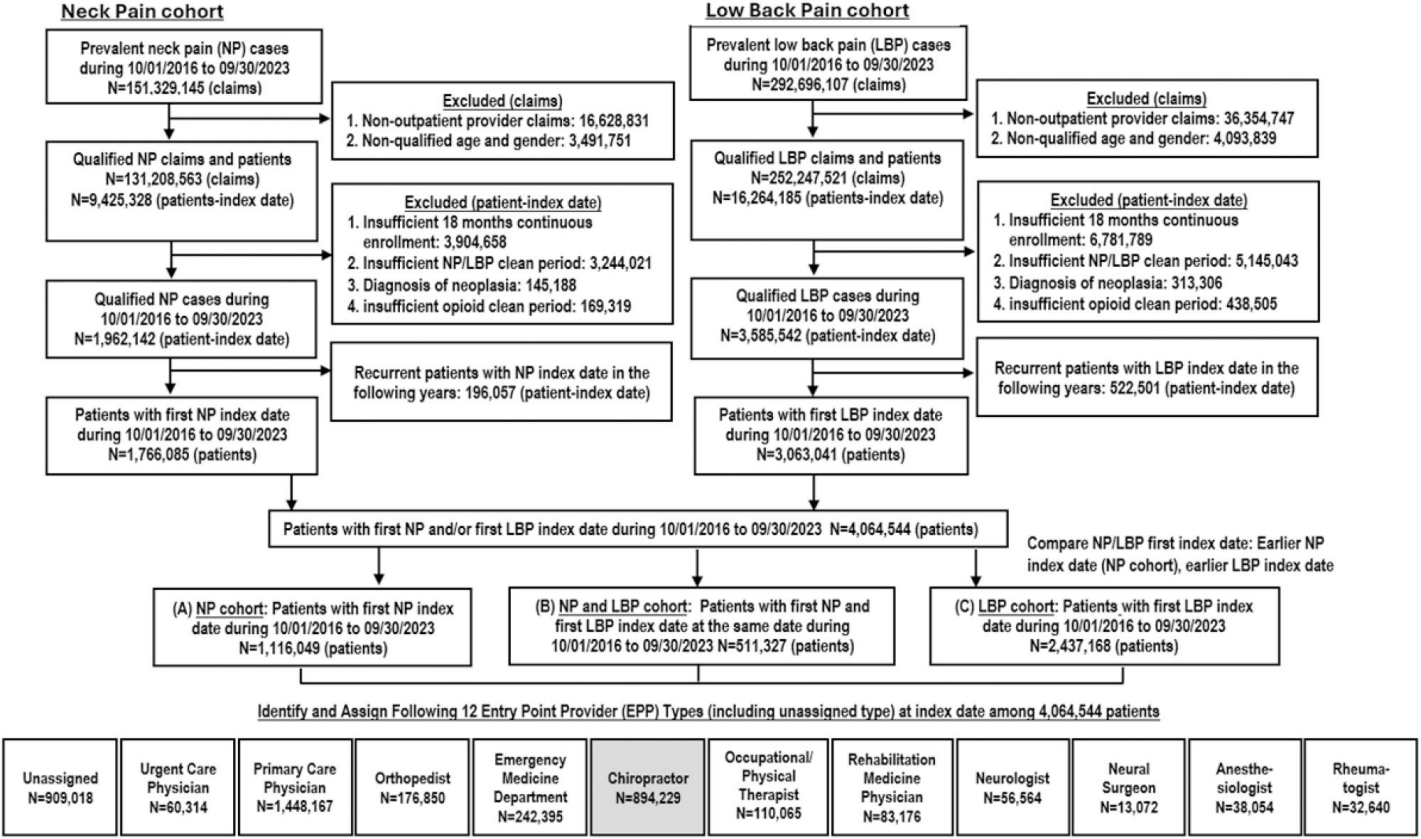
Cohort design.

We obtained data from October 2016 to September 2023 for adults (18-89 years old) from the Optum Labs Data Warehouse (OLDW) which contains de-identified administrative claims data, including medical and pharmacy claims and eligibility information for about 350 million enrollees, representing a mix of ages and regions across the United States.^21^ This analysis was an expansion of our previous study on neck pain.^3^ The design of our retrospective cohort is detailed in Figure 1. Clinical conditions and events were identified using International Classification of Diseases, Tenth/Ninth Revision, Clinical Modification (ICD-10-CM) diagnosis codes or Current Procedural Terminology (CPT) procedure codes. Comorbidities were identified using the AHRQ Elixhauser Comorbidity Software.^22^

Eligibility criteria included—(1) continuous enrollment with medical and pharmacy coverage for at least 18 months, including 12 months before and at least 6 months after the index visit; (2) absence of any visit for NP/LBP or injury (clean period); (3) absence of opioid prescription for 30 days before index visit; (4) no diagnosis of neoplasia; and (5) index visit was to a chiropractor. Our final analytical cohort included 894 229 patients. These patients belonged to 1 of 3 clinical subcohorts based on their diagnosis of NP/LBP on the index visit: (1) Cohort 1—NP alone; (2) Cohort 2—LBP alone; and (3) Cohort 3—both NP and LBP.

These patients were assigned the following provider types/categories throughout their care trajectory—chiropractor, primary care (PC) (including family practice and internal medicine), emergency medicine (EM), orthopedics, physical/occupational therapy (OT/PT), neurology, neurosurgeon, rheumatologist, anaesthesiologist, rehabilitation medicine, urgent care, or unassigned using the provider specialty codes on physician claims. When a claim had multiple provider types on the index date, we prioritised PC, and physician or chiropractor over OT/PT. For chiropractic visits, we assessed the occurrence of mutually exclusive CPT codes for chiropractic manipulative treatment (CMT): 98940 (1-2 spinal regions), 98941 (3-4 spinal regions), 98942 (5 spinal regions), and 98943 (extraspinal).

### Methods

We used an R package spectral clustering (SC) algorithm (Spectrum)^23^ to group patients based on their chiropractic visits using default options and FASP=TRUE for Fast Approximate Spectral Clustering^24^ (run times ranging from 20 to 30 minutes). The input to SC was the number of visits to the chiropractor by week for 6 months (weeks 1-26) for each patient. Spectral graph theory associates the eigenvalues of a matrix to the properties of a graph via the Laplacian matrix.^25^^,^^26^ It operates on graphs that are constructed between neighbouring nodes that represent data points (ie, patients). Eigenvectors identify arbitrarily shaped clusters (with convex or non-convex boundaries).^27–30^ Spectral clustering is robust to outliers, and does not require the pre-specification of number of clusters as in K-means or latent class analyses.^31–33^ Since our aim was to discover novel patterns, the way to implement this is unsupervised machine learning with the fewest assumptions imposed on the data structure such as the number of clusters, cluster shape, complete and large separation versus overlapping clusters, sparse vs dense clusters, etc.^7^^,^^30^^,^^34^^,^^35^ The spectral clustering pipeline involves (1) creation of the similarity matrix, then (2) creation of the Laplacian matrix, and finally (3) creation of clusters.^30^^,^^36^ The final clusters are plotted in 2-D using the first 2 principal components. We did not use the eigen gap-statistic to determine the number of clusters as it was not essential for us to restrict the number or size of clusters. The eigen gap heuristic does not work well if there are overlapping clusters, which is true in real-world datasets.^7^^,^^30^^,^^34^^,^^37^

We sought to identify and explore any distinguishing characteristics (pairwise cluster comparisons) across the patient clusters.^11^ To that end, we applied EBM to assess feature importance (feature ranking) and compare it with Hierarchical Group-LASSO Regularisation (HGLR) to select features with the outcome being the cluster label. EBM is a tree-based gradient boosting Generalised Additive Model (GAM) with advantages of interpretability.^38^^,^^39^ GAMs are statistical models that extend the generalised linear models (GLMs)^40^ to include non-linear relationships and non-Gaussian response distributions.^38^ The following equations show the models in order of their emergence. The progressive changes were made to allow for greater flexibility in determining how the outcome depends on the predictors, interpretability and calculation of the shape functions.


(1a) $$g\left(E\left[Y|x\right]\right)=\beta_{0}+\sum_{i=1}^{p}\beta_{i}x_{i}\;\;(\mathrm{GLM},\;\mathrm{Nelder}-\mathrm{Wedderburn}\;1972)$$

(1b) $$g\left(E\left[Y|x\right]\right)=\beta_{0}+\sum_{i=1}^{p}f_{i}\left(x_{i})\;\;(\mathrm{GAM},\;\mathrm{Hastie}-\mathrm{Tibshirani}\;1986\right)$$

(1c) $$g\left(E\left[Y|x\right]\right)=\sum_{i=1}^{p}f_{i}\left(x_{i}\right)+\sum_{i,j=1}^{p}f_{\mathrm{ij}}\left(x_{i},x_{j}\right)(i\neq j)\;\;({\mathrm{GA}}^{2}M,\;\mathrm{Lou}\;2013)$$

(1d) $$g\left(E\left[Y|x\right]\right)=\beta_{0}+\sum_{i=1}^{p}f_{i}\left(x_{i}\right)+\sum_{i,j=1}^{p}f_{\mathrm{ij}}\left(x_{i},x_{j}\right)(i\neq j)\;\;(\mathrm{EBM},\;\mathrm{Nori}\;2019)$$

where *g*(.) is the link function that links the random component (E[Y—x]) to the systematic component (the linear predictors), *Y* is the exponential family distribution, *β* is set of regression coefficients estimated from the data using maximum likelihood estimates (MLE) and *f_i_* is the shape function or feature function. *Y* refers to binary outcomes/classes in classification settings.

Lou et al. introduced systematic search for pairwise interaction terms (*GA*^2^*M)* and tree-based methods for calculating the feature functions. EBMs used gradient boosting to tune the feature functions, a low learning rate, inner/outer bagging for greater accuracy, estimated standard errors. EBM estimates each feature function (using piecewise constant functions as in decision trees) in a round-robin fashion. The feature rank plots show the mean absolute contribution (importance score) used to order features in the training set. Essentially, the weights of the individual features are summed (and averaged over the training set) to make a prediction.^41^ This global importance score is based on the sample weight and number of non-zero valued records in either class (class refers to the dichotomous outcomes), therefore, it is not to be interpreted as being more predictive of a single class, say, the positive group (class 1). Therefore, if *p* features are input into the EBM, it will output a minimum of *p* but possibly ≥ *p* features as they include some interactions. However, one can choose a subset from this ordered set of features that can be fed into a penalised regression feature selection model to get a parsimonious set. The selected features can be put into interpretable parametric models (eg, logistic/linear regression) to obtain parameter estimates (magnitude and direction). The goal of GAM is to model complex non-linear relationships through smooth, flexible functions. EBMs have been applied for predicting 30-day hospital readmission, risk of pneumonia, and outcomes of treatment for COVID-19, etc.^20^^,^^42^ We used the python package InterpretML^41^^,^^43^ to implement EBM (with default options and fitted 2 EBMs with different random seeds) to explore cluster characteristics and assess feature importance. We removed variables with low rank to reduce the computational burden in the feature selection phase. EBM run times were 10-30 minutes.

For data with *n* observations and *p* features, the number of possible models is 2^*p*^. Stepwise approaches have serious limitations^44–48^ and, hence, the need for penalised regression methods, which is the process of shrinking coefficient estimates towards zero.^49^^,^^50^ Ridge regression provides a unique solution,^50^ however, its main drawback is that it retains all the predictors. Least absolute shrinkage and selection operator (LASSO)^49^ provides parsimonious models via penalised regression. However, its main drawbacks are: (1) In the *p > n* case, the LASSO selects a maximum of *n* variables; (2)If there is a set of collinear variables, LASSO arbitrarily picks one of them; (3) Solutions are not unique as the LASSO criterion is not strictly convex. This can lead to instability and poor generalisation; and (4) LASSO does not automatically identify all pairwise interactions but rather depends on manual specification of each interaction. This leads to spurious interactions being forced in or important interactions being omitted. Elastic net combines the advantages of LASSO and ridge regression.^51^ However, as it combines LASSO and ridge regression and introduces the *l*1 ratio, which is determined through cross-validation (CV), it is computationally intensive.

Hierarchical Group-LASSO Regularisation (HGLR) is a feature selection method for identifying interactions.^12^^,^^52^ It is given by:


(2) $$\underset{µ,\beta }{\text{argmin}}\frac{1}{2}∥Y-µ\cdot 1-X_{1}\beta_{1}-X_{2}\beta_{2}-X_{1:2}\beta_{1:2}{∥}_{2}^{2}+\lambda (∥\beta_{1}{∥}_{2}+∥\beta_{2}{∥}_{2}+∥\beta_{1:2}{∥}_{2})$$

*Y* ∈ R^*n*^: Response vector of *n* observations
*µ* ∈ R: Intercept parameter
**1** ∈ R^*n*^: Vector of ones
*X*_1_ ∈ R^*n×L*^1: Feature matrix for variable 1 (matrix for categorical variables with *L*_1_ levels)
*X*_2_ ∈ R^*n×L*^2: Feature matrix for variable 2 (matrix for categorical variables with *L*_2_ levels)
*X*_1:2_ ∈ R^*n×*^^(^^*L*^1 *×L*2): Interaction matrix, computed as *X*_1_ ∗ *X*_2_ (element-wise product)
*β*_1_ ∈ R^*L*^1: Main effect coefficients for variable 1
*β*_2_ ∈ R^*L*^2: Main effect coefficients for variable 2
*β*_1:2_ ∈ R^*L*^1 *×L*2: Interaction coefficients between variables 1 and 2
*λ >* 0: Regularization parameter controlling the amount of penalisation
∥ · ∥_2_: *L*_2_ norm (Euclidean norm), $||v|{|}_{2}\;=\;\sqrt{\sum_{i}\;v_{i}^{2}}:$ measures the length or magnitude of the vector

The group-lasso penalty enforces strong hierarchy: if *β*_1:2_ ≠ 0 (interaction is included), then both *β*_1_ ≠ 0 and *β*_2_ ≠ 0 (both main effects are also included).^12^

HGLR is effective when the number of covariates is high and may include interactions.^13^^,^^53–55^ HGLR is an explainable model that sets up main effects and interactions and then performs feature selection via group-LASSO.^56^ Using R package glinternet^57^ we implemented a classification model with family=binomial, 5-fold CV and 80 cores for parallel processing. Run times ranged from 2 to 10 hours.

### Analyses

The 77 input baseline features (since they occurred on index visit or within the first 30 days) to the EBM included age, gender, sub-cohort, prescriptions for muscle relaxant or benzodiazepines or opioids within the first 30 days of their index visit, imaging within the first 30 days of their index visit, several clinical diagnosis recorded on the index visit, counts of prior visits to each of the provider types, indicator of concurrent drugs prescriptions, Elixhauser scores, diagnosis of complications of spinal manipulation or spinal surgery (Supplementary File 1). A reduced set of 37 input features to the HGLR overlapped with above list with the exception of Elixhauser scores, indicator of concurrent drugs prescriptions, and diagnosis of complications of spinal manipulation/surgery, which were dropped due to EBM’s low ranking. HGLR and EBM models were run on 80-20% training-test split data. We used area under the receiver operating characteristic (AUC or C-Statistic) and the area under the precision-recall curve (AUPRC) as measures of discrimination on imbalanced datasets. We chose the HGLR model with regularisation parameter (lambda) set to lambda1se (minimum lambda value + 1 standard deviation for best generalisability) on the training set, following machine learning protocols.^13^^,^^57^ Finally, a logistic regression model was used to view the association between the HGLR selected features and the cluster label. Although there were only 37 covariates, the number of models with different combinations of these covariates is exponential, hence, feature selection is necessary. Feature selection helps create generalisable parsimonious models whose parameter estimates and standard errors are robust. Variance inflation factor (VIF) was calculated to check for multicollinearity to ensure robust estimates interpretation of the predictors.^58^

The analytical pipeline was: (1) apply spectral clustering on patient-level data using number of weekly visits to the chiropractor (sub-population identification phase); then (2) use EBM to identify features that were important to understand the sub-populations (feature ranking); then (3) perform feature selection using HGLR on the reduced set of features highlighted by EBM (feature selection) to compare sub-populations; and (4) use logistic regression to obtain parameter estimates of the HGLR selected features.

## Results

In this longitudinal population (*N* = 4 064 544) with an initial diagnosis of low back pain (LBP), neck pain (NP), or both, the largest proportion of patients were first seen by a primary care (PC) physician (45.9%), followed by chiropractors (28.3%) as shown in Figure 1. Among patients with NP or LBP alone, the plurality were first seen by PC (43.7% and 55.1%, respectively) followed by chiropractors (24.9% and 18.2%) and emergency medicine physicians (EmrMed) (9.9% and 7.4%). By contrast, among patients with an initial diagnosis of both NP and LBP, chiropractors initiated treatment for 77.1%. The overall frequency of chiropractic care among patients with NP or LBP in our sample (28.3%) was very similar to that reported in national samples such as the Medical Expenditure Panel Survey (27.8%).^59^

**Figure 2:**
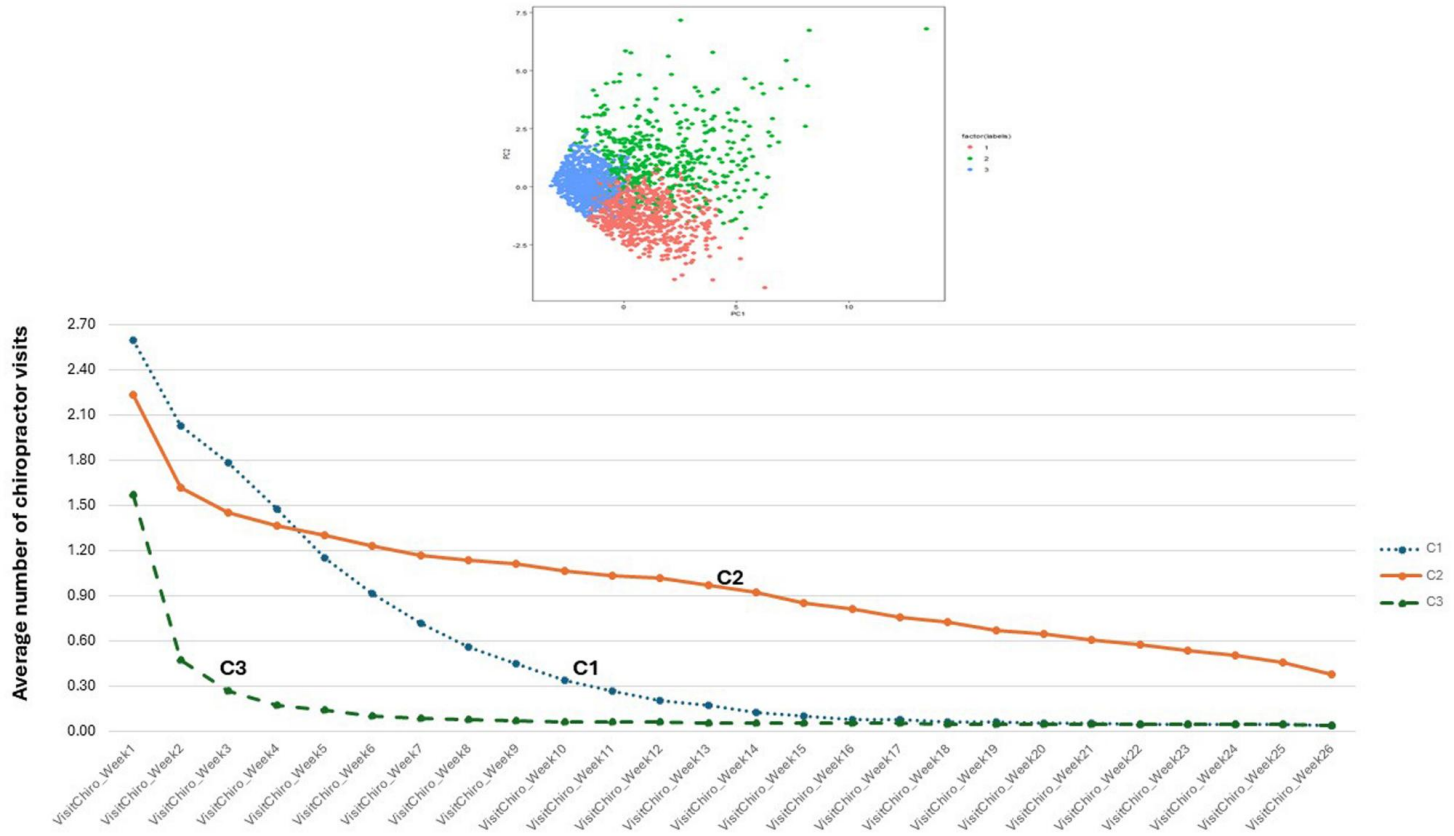
(Top) Spectral clustering—3 patient sub-populations. The plot is shown with data represented by—principal component 1 (x-axis) and principal component 2 (y-axis); (Bottom) Plots of the average number of visits to the chiropractor (Y-axis) by week (X-axis) from the initial/index visit for all 3 clusters.

Further analyses were limited to 894 229 individuals (mean age 47.2 [± 17.2] years old; 52.7% female; 66% White race/ethnicity) who saw a chiropractor on the index visit (Table 1). Spectral clustering (SC) using the number of visits to a chiropractor in weeks 1-26 (6-month period) resulted in 3 patient clusters/groups (C): cluster 1 with 149 025 patients (16.7%), cluster 2 with 38 913 patients (4.4%) and cluster 3 with 706 291 patients (79.9%) (Figure 2). While the index visit was with a single provider according to the cohort definition, follow-up visits could involve other or multiple providers. The average number of visits to any chiropractor by week is shown in Figure 2.

**Table 1:** Patient characteristics in cohort.

Characteristics	Overall				Within clusters			
	*N*	%	Cluster 1	%	Cluster 2	%	Cluster 3	%
Total patients	**894** **229**	100.00	**149** **025**	100.00	**38** **913**	100.00	**706** **291**	100.00
**Initial diagnosis on index visit (clinical cohort)**								
Neck pain (NP)	196799	22.01	32594	21.87	8120	20.87	156085	22.10
Low back pain (LBP)	346697	38.77	58199	39.05	13164	33.83	275334	38.98
NP & LBP	350733	39.22	58232	39.08	17629	45.30	274872	38.92
**Sex**								
Male	422962	47.30	65610	44.03	15898	40.86	341454	48.34
Female	471267	52.70	83415	55.97	23015	59.14	364837	51.66
Commercial insurance	721279	80.66	116378	78.09	29039	74.63	575862	81.53
**Race**								
Asian	24728	2.77	4086	2.74	1284	3.30	19358	2.74
Black	39234	4.39	7144	4.79	1994	5.12	30096	4.26
Hispanic	59529	6.66	9284	6.23	2428	6.24	47817	6.77
White	591454	66.14	99292	66.63	25674	65.98	466488	66.05
Unknown	179284	20.05	29219	19.61	7533	19.36	142532	20.18
Recurrent patient^a^	144306	16.14	21773	14.61	4289	11.02	118244	16.74
**Spine-related comorbidities on index** ^b^	159910	17.88	35830	24.04	9038	23.23	115042	16.29
Spinal radiculopathy	134247	15.01	30510	20.47	7721	19.84	96016	13.59
Headache	26173	2.93	5558	3.73	1391	3.57	19224	2.72
**Imaging 0-30 days from index** ^c^								
X-ray on cervical region	104583	11.70	37325	25.05	9754	25.07	57504	8.14
X-ray on non-cervical region	149265	16.69	51459	34.53	12387	31.83	85419	12.09
CT on cervical region	968	0.11	155	0.10	49	0.13	764	0.11
CT on non-cervical region	783	0.09	114	0.08	28	0.07	641	0.09
MRI on cervical region	3109	0.35	796	0.53	178	0.46	2135	0.30
MRI on non-cervical region	7038	0.79	1771	1.19	350	0.90	4917	0.70
Continuous variables	Mean	±Std	Mean	±Std	Mean	±Std	Mean	±Std
**Age**	47.23	17.24	48.96	17.18	49.86	17.33	46.72	17.21
**Number of prior visits to**								
PCP	2.90	3.88	3.08	4.15	3.28	4.35	2.84	3.80
Emergency Department Physician	0.20	0.74	0.20	0.79	0.21	0.83	0.19	0.72
Anesthesiologist	0.17	0.53	0.18	0.54	0.20	0.60	0.16	0.52
Chiropractor	0.09	1.05	0.05	0.81	0.21	2.52	0.09	0.95
Neurologist	0.09	0.57	0.10	0.59	0.12	0.77	0.08	0.55
Neurosurgeon	0.00	0.10	0.00	0.12	0.00	0.10	0.00	0.10
Orthopedist	0.26	1.21	0.29	1.31	0.34	1.52	0.25	1.17
Occupational/Physical Therapist	0.54	3.11	0.64	3.44	0.89	4.43	0.50	2.94
Rehabilitation Medicine Physician	0.05	0.57	0.06	0.66	0.07	0.74	0.05	0.53
Rheumatologist	0.03	0.37	0.04	0.42	0.05	0.44	0.03	0.36
Urgent Care Physician	0.09	0.41	0.09	0.41	0.11	0.47	0.09	0.41

a Recurrent Patient refers to situation where the patient had no visits related to NP/LBP for 12 months and then returned to see a chiropractor with NP/LBP diagnosis. They would still have to meet the inclusion/exlusion criteria to enter the analytical cohort as outlined.

b Spine-related comorbidities on index includes the following conditions: Myelopathy, Spinal radiculopathy, Cauda equina syndrome and headache. Myelopathy and Cauda equina syndrome data are not shown due to small cell size policy.

c Imaging variables refers to CT, Xray, MRI performed on certain regions of the spine-cervical (NP diagnosis)/non-cervical (LBP diagnosis).

**Figure 3:**
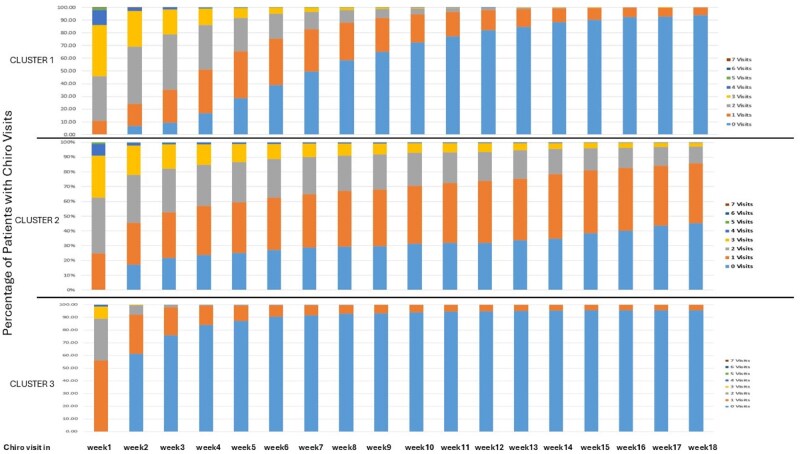
Percentage of patients with visits (or no visits) to the chiropractor by week and cluster.

Figure 3 shows the percentage of patients with 0-7 chiropractor visits for the first 18 weeks.

The 3 clusters showed very different trajectories of chiropractic visits. The majority (79%) of patients were in cluster 3, which rapidly tapered from a mean of 1.6 visits in week 1, to 0.5 visits in week 2, to 0.1 visits by week 12.

Cluster 2 was the smallest group (4%) and had the highest percentage of patients who received combined diagnosis of NP/LBP on their index visit, along with the most persistent utilisation of chiropractic services. Figure 3 highlights that over half of patients in cluster 2 had one or more weekly visits even at 4.5 months after the index visit, whereas patients in clusters 1 and 3 usually discontinued chiropractic care within 2-3 months. Given these patterns of utilisation, we labelled the clusters based on their treatment patterns as: cluster 1—moderate dose therapy, cluster 2—high dose, and cluster 3—low dose. Associated symptoms of spinal radiculopathy and headache were similarly prevalent in clusters 1 and 2, but less prevalent in cluster 3 (Table 1). Analyses of income, education and home ownership did not show notable differences across clusters (data not shown). Recurrence of NP and/or LBP after a clean period of at least 12 months was more frequent in clusters 1 (14.6%) and 3 (16.7%) than in cluster 2 (11.0%), suggesting that some patients re-initiate chiropractic care when their symptoms recur after a long period of remission.

**Figure 4:**
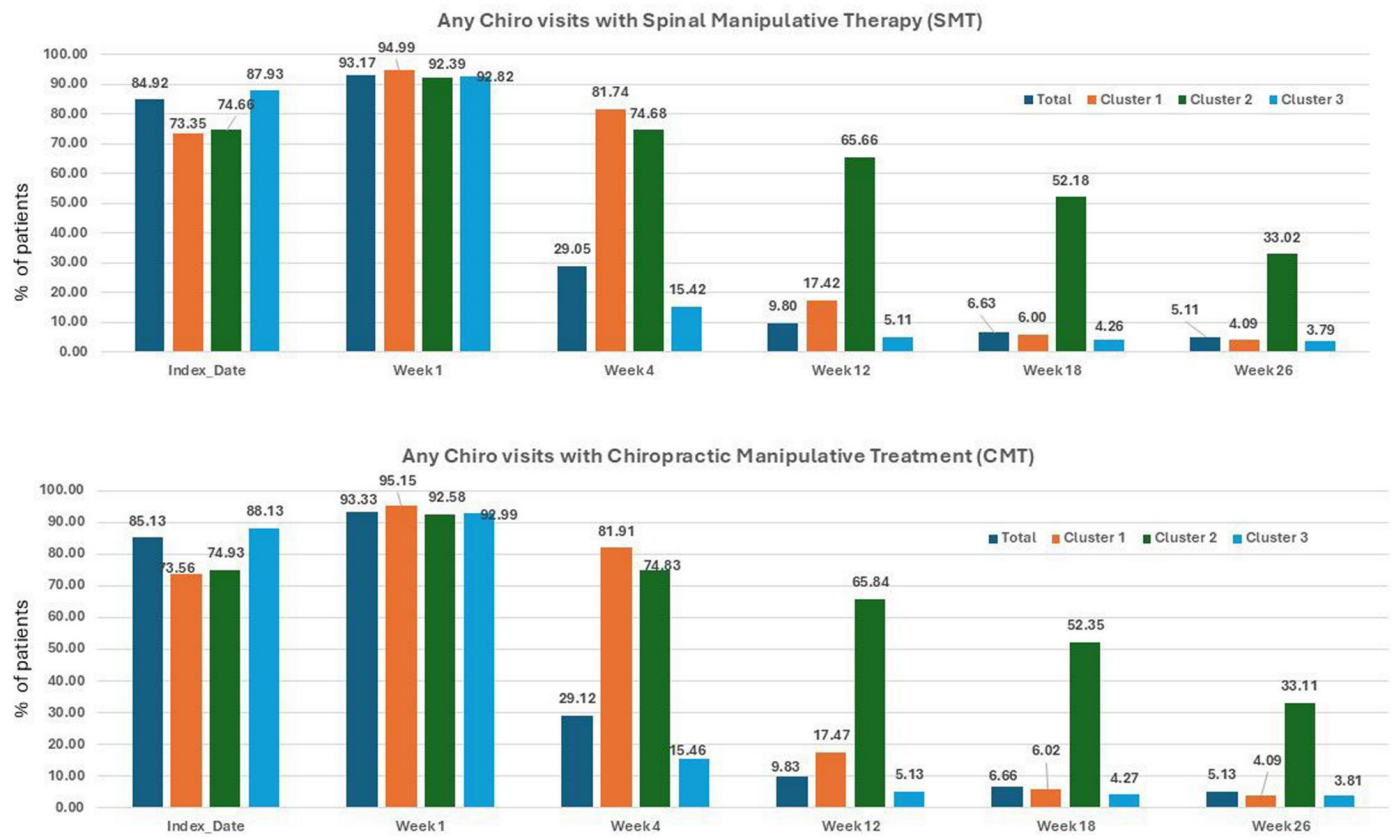
Percentage of patients with spinal manipulative therapy (SMT) or chiropractic manipulative treatment (CMT).

**Figure 5:**
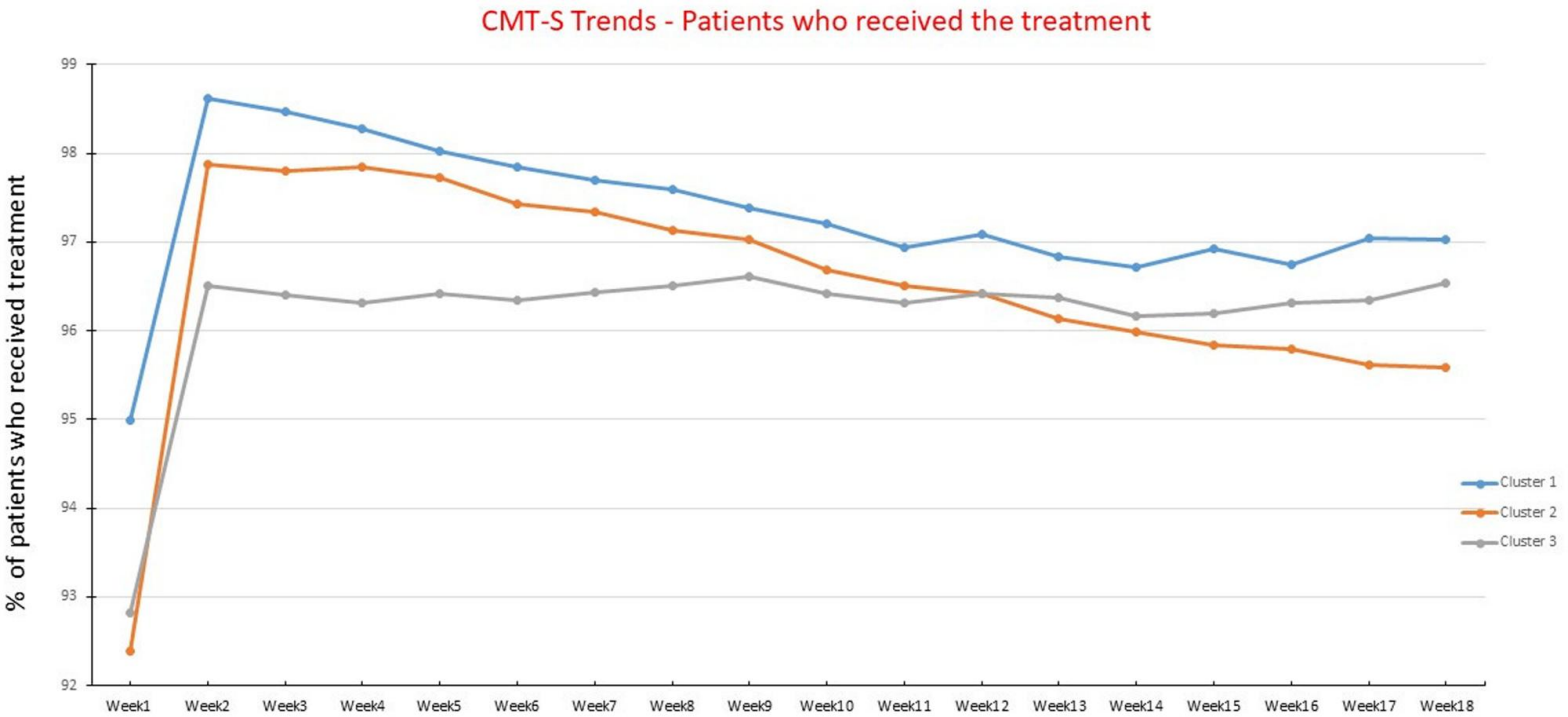
Percentage of patients who visited a chiropractor and received spinal CMT (CMT-S).

Across all clusters, over 73% of patients received spinal CMT on their index visit, and over 92% received spinal CMT by the end of week 1 (Figure 4). Values were minimally higher when extraspinal CMT was included. Nearly all (*>*95%) who remained in chiropractic care received spinal CMT in subsequent visits (Figure 5), with no significant trends or between-cluster differences in the use of spinal CMT. Specifically, in cluster 3, 39% of patients had 2 or more episodes of spinal CMT in week 1, versus 80% and 66% in clusters 1 and 2, respectively. By week 3, only 9% of cluster 3 patients who remained in chiropractic care received 2 or more episodes of spinal CMT, versus 70% and 59% of patients in clusters 1 and 2, respectively.

Table 2 shows the time to discontinuation of chiropractor visits and intensity of treatment until discontinuation. Over 90% of patients in cluster 3 discontinued chiropractic care within 2 weeks and 3 visits from the index date. Clusters 1 and 2 showed more similar patterns, with a mean of 10.3 visits over 5.3 weeks for cluster 1 versus 12.1 visits over 6.7 weeks for cluster 2. However, cluster 2 had a markedly skewed distribution, as 5% of its patients had at least 34 visits over at least 17 weeks.

**Table 2:** Descriptive analyses of time to discontinuation of chiropractor visits.

Cluster	No. patients	Variable description	Mean	90th Pctl	95th Pctl	99th Pctl
1	149 025	No. weeks from index date to discontinuation	5.30	7	11	13
		Total no. visits from index date to discontinuation	10.26	13	21	26
2	38 913	No. weeks from index date to discontinuation	6.66	10	17	23
		Total no. visits from index date to discontinuation	12.14	18	34	45
3	706 291	No. weeks from index date to discontinuation	1.68	2	4	6
		Total no. visits from index date to discontinuation	2.35	3	6	7

**Figure 6:**
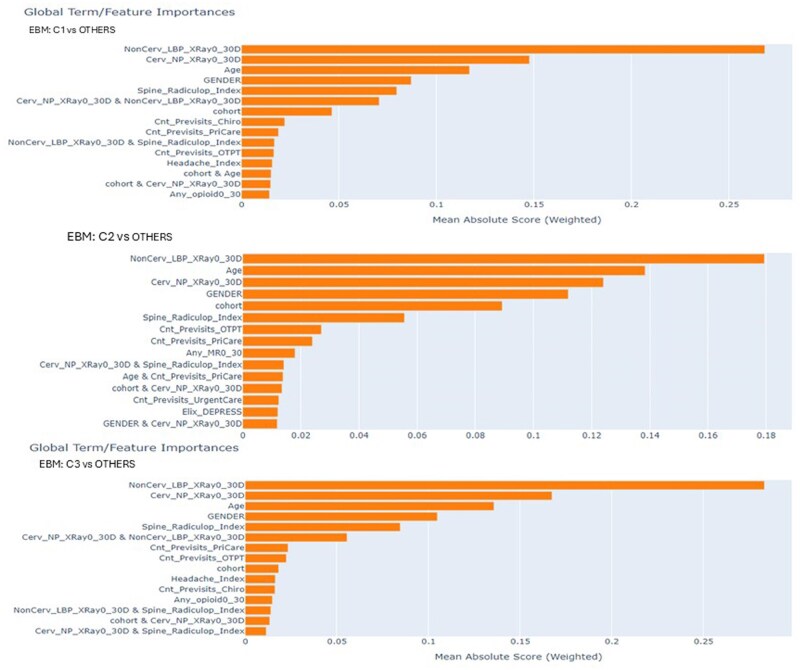
Feature ranking using EBM while comparing: (top-a) Cluster 1 vs others; (middle-b) Cluster 2 vs others; and (bottom-c) Cluster 3 vs others; X-axis shows the mean absolute importance for each feature which corresponds to its impact on the prediction (on either class 0/1) in the training set.

For clarity, we show the top 15 highly ranked features across the patient clusters in Figure 6. EBM identifies how much a feature contributes to one cluster vs another and ranks them. Table 3 shows the covariate-adjusted estimates of HGLR selected features where the outcome was defined as a cluster label. Fully saturated models with the 37 main effects resulted in models either not converging or extremely large standard errors and z-scores.

**Table 3:** Parameter estimates of the HGLR selected covariates in the logistic regression models.

Variables	Estimates	Risk ratio	2.5 %	97.5 %
**Model 1: Moderate (ref.) vs high dose**				
BothDx Index⋄	0.1810 (±) 0.0090	1.198	1.174	1.223
Disloc SpineInj Index⋄	0.2540 (±) 0.0970	1.289	1.039	1.600
Cerv NP XRay 0 30D⋄	−0.2660 (±) 0.0200	0.767	0.735	0.800
NonCerv LBP XRay 0 30D⋄	−0.2810 (±) 0.0140	0.755	0.732	0.778
Age⋄	0.0020 (±) 0.0000	1.002	1.002	1.003
Cnt Previsits PriCare⋄	0.0050 (±) 0.0010	1.005	1.003	1.007
Cnt Previsits Chiro⋄	0.0280 (±) 0.0040	1.029	1.026	1.032
Cnt Previsits OTPT⋄	0.0100 (±) 0.0010	1.010	1.008	1.012
BothDx Index * Disloc SpineInj Index⋄	−0.3660 (±) 0.1940	0.693	0.451	1.064
Cerv NP XRay 0 30D * NonCerv LBP XRay 0 30D⋄	0.5550 (±) 0.0260	1.742	1.647	1.842
**Model 2: Low (ref.) vs high dose**				
GENDER⋄	0.3244 (±) 0.0116	1.3833	1.3512	1.4160
Cerv NP XRay 0 30D⋄	1.0112 (±) 0.0251	2.7489	2.6091	2.8962
Any MR 0 30⋄	−0.2890 (±) 0.0502	0.7490	0.6781	0.8274
NonCerv LBP XRay 0 30D⋄	0.8980 (±) 0.0153	2.4546	2.3794	2.5323
Spine Radiculop Index⋄	0.3434 (±) 0.0147	1.4097	1.3685	1.4523
NonCerv LBP MRI 0 30D	0.4864 (±) 0.0772	1.6264	1.3914	1.9012
Age⋄	0.0117 (±) 0.0003	1.0117	1.0112	1.0123
Cnt Previsits Chiro	0.0443 (±) 0.0070	1.0453	1.0420	1.0486
Cnt Previsits OTPT⋄	0.0215 (±) 0.0012	1.0218	1.0198	1.0237
GENDER * Cerv NP XRay 0 30D⋄	−0.1274 (±) 0.0221	0.8804	0.8403	0.9224
Any MR 0 30 * NonCerv LBP XRay 0 30D	−0.4838 (±) 0.0772	0.6165	0.5279	0.7199
Cerv NP XRay 0 30D * NonCerv LBP XRay 0 30D	−0.1963 (±) 0.0270	0.8218	0.7772	0.8689
Cerv NP XRay 0 30D * Spine Radiculop Index⋄	−0.2799 (±) 0.0261	0.7559	0.7153	0.7988
NonCerv LBP XRay 0 30D * NonCerv LBP MRI 0 30D	−0.8913 (±) 0.1047	0.4101	0.3314	0.5075
NonCerv LBP XRay 0 30D * Cnt Previsits Chiro	−0.0323 (±) 0.0117	0.9682	0.9477	0.9891
**Model 3: Low (ref.) vs moderate dose**				
GENDER⋄	0.1808 (±) 0.0059	1.1982	1.1829	1.2137
Age⋄	0.0077 (±) 0.0001	1.0077	1.0074	1.0080
Any MR 0 30⋄	0.0488 (±) 0.0207	1.0500	1.0045	1.0975
Any opioid 0 30⋄	−0.2966 (±) 0.0266	0.7433	0.7019	0.7872
Cerv NP XRay 0 30D⋄	1.0418 (±) 0.0106	2.8344	2.7641	2.9064
NonCerv LBP XRay 0 30D⋄	1.0609 (±) 0.0066	2.8890	2.8449	2.9337
Cnt Previsits PriCare⋄	0.0050 (±) 0.0005	1.0050	1.0038	1.0061
Cnt Previsits OTPT⋄	0.0094 (±) 0.0006	1.0094	1.0080	1.0108
Spine Radiculop Index⋄	0.3830 (±) 0.0099	1.4666	1.4348	1.4992
Headache Index⋄	0.2616 (±) 0.0146	1.2990	1.2576	1.3418
GENDER * Any opioid 0 30	−0.0450 (±) 0.0359	0.9560	0.8846	1.0331
GENDER * Cerv NP XRay 0 30D	−0.0700 (±) 0.0098	0.9324	0.9106	0.9548
GENDER * Spine Radiculop Index	−0.0557 (±) 0.0110	0.9458	0.9221	0.9701
Any MR 0 30 * NonCerv LBP XRay 0 30D⋄	−0.4140 (±) 0.0297	0.6610	0.6191	0.7057
Cerv NP XRay 0 30D * NonCerv LBP XRay 0 30D⋄	−0.7440 (±) 0.0108	0.4752	0.4629	0.4878
Cerv NP XRay 0 30D * Spine Radiculop Index⋄	−0.1634 (±) 0.0119	0.8492	0.8244	0.8748
NonCerv LBP XRay 0 30D * Spine Radiculop Index⋄	−0.1785 (±) 0.0114	0.8365	0.8136	0.8600
NonCerv LBP XRay 0 30D * Headache Index	−0.1281 (±) 0.0236	0.8797	0.8296	0.9329

Variables marked with an ⋄ refer to those that were also present in EBM’s top 20 variables list.

Disloc SpineInj Index: Dislocation spinal injury diagnosis on index visit; Spine Radiculop Index: spinal radiculopathy diagnosis on index visit; Headache Index: headache diagnosis on index visit; BothDx Index: Indicator of whether a single or both NP/LBP diagnoses were given. Value = 1 (concurrent diagnosis) refers to patients who received both NP and LBP diagnoses on index visit; Cerv NP XRay 0 30D: Cervical NP Xray performed within 0-30 days from index date. Similar definition for MRI and LBP; NonCerv LBP XRay 0 30D: NonCervical LBP Xray performed within 0-30 days from index date. Similar definition for MRI and LBP; Any MR 0 30: Any muscle relaxant prescribed within 0-30 days from index date. Similar definition for opioid; GENDER: coded as Male= 0 and Female =1; Cnt Previsits PriCare or OTPT or Chiro: Count of visits to Primary Care or occupational/physical therapists or chiropractors before index visit with new NP/LBP diagnosis.

## Discussion

Neck pain and low back pain are high prevalence conditions with substantial impact on healthcare spending, patient functioning, and quality of life. Patients with these conditions often seek chiropractic care, which entails multiple services including spinal manipulation. Randomized controlled trials and systematic reviews have supported multi-modal chiropractic care, including spinal manipulation, yet little is known about longitudinal patterns of chiropractic care in the US. Also, the optimal dosing for initial NP or LBP presentations has not been established. We used unsupervised ML methods in a large national claims data warehouse to describe longitudinal patterns of insurance-covered chiropractic care, for the first time, with the objective of informing future comparative effectiveness and cost-effectiveness research.

Guidelines largely based on expert opinion have recommended spinal CMT 2-3 times per week for 2-4 weeks as an initial trial period for acute and subacute low back pain.^60^^,^^61^ In this analysis, cluster 1 (with 16.7% of all patients who received chiropractic care for NP or LBP) demonstrated a guideline-concordant treatment pattern, with a mean of 10.3 visits over 5.3 weeks, and over 60% of patients receiving 2-3 visits during each of the first 3 weeks. According to guidelines, cluster 3 (with 79.9% of patients) may have been undertreated, with a mean of only 2.3 chiropractor visits over 1.7 weeks. Cluster 2 raises important questions about overtreatment, as the majority of its patients continued having at least one weekly visit through the 18th week and beyond (and 5% had at least 34 visits in total). This was an unexpected discovery as no study has previously identified this longitudinal pattern. To gather more insights, we compared patient characteristics and initial diagnostic and treatment choices across clusters. Notably, the high-dose cluster 2 was similar to moderate-dose cluster 1 with respect to the prevalence of spinal radiculopathy or headache, initial imaging evaluation, and provider visit patterns before the index visit.

Due to the large number of features and size of data, feature selection using HGLR, allowing for all combinations of first order interactions, can take several days on a standard systems (less time on advanced systems). While robust and accurate, such precision is unnecessary for exploratory analyses. Therefore, we tried a novel technique by applying EBM to rank features based on their ability to differentiate clusters and then fed a subset of highly ranked features into HGLR. This analytic approach illuminated the covariates associated with the dose of chiropractic services. The VIFs of the predictors in all models were less than 5 indicating acceptable values for robust interpretation of estimates. EBM showed imaging variables as highly ranked features distinguishing clusters, suggesting either that chiropractors’ imaging preferences are correlated with their longitudinal treatment preferences, or that their use of diagnostic imaging is a proxy for unmeasured severity of pain or functional limitations.

Parameter estimates of the HGLR selected covariates are shown in Table 3. Both HGLR and EBM highlighted that the count of prior visits to the chiropractor, OT/PT and PC were important indicators of dose, suggesting that patients may have an affinity for certain providers with certain treatment preferences. Table 3 shows:

- Model 1 Moderate dose (reference) vs high dose: a combined diagnosis of NP and LBP, and a diagnosis of dislocation spinal injury at the index visit, were associated with a higher dose of chiropractic care. Patients in the high dose cluster had more visits to chiropractors for other reasons in the year prior to the index visit. Gender (not selected by HGLR) and age were not associated with dose of therapy.
- Model 2 Low dose (reference) vs high dose: female gender, increased age, and a diagnosis of spinal radiculopathy on the index date were associated with a higher dose of chiropractic therapy. Patients in the high-dose cluster were also less likely to have filled a muscle relaxant prescription within 30 days of their index visit, suggesting differences in how they use non-chiropractic providers for their NP and LBP. Furthermore, the use of imaging services was higher in the high dose group compared to those in the low dose group.
- Model 3 Low dose (reference) vs moderate dose: Low and moderate dose therapy clusters differed in the prevalence of a concurrent diagnosis of spinal radiculopathy and headache on their index visit. Interestingly, opioids were an important factor in that patients in the moderate-dose cluster 1 were less likely to be prescribed opioid analgesics than patients in low-dose cluster 3, suggesting that a moderate dose of SMT may reduce demand for opioid prescriptions. Also, the early use of imaging services was higher in the moderate dose group.

It is valid to include visits and patient characteristics as input into SC, leading to greater model complexity and uninterpretable results. To aid explainability at every step, we kept the analyses of weekly visits separate from those of patient characteristics, ie, split the task to reduce problem complexity. We wanted to showcase the performance of EBM compared to HGLR which is a more complex, and computationally intensive feature selection method. EBMs would enable researchers to quickly identify important features more systematically than outdated methods for para-metric effect models.^14^^,^^62–66^ Results show that EBMs are effective tools for identifying features, albeit, a minimum threshold needs to be set to decide the final number of features. As we used EBM as a feature ranking tool, it ranks all the features according to an importance score. The advantage of this is that a researcher can use a lowly ranked feature, if there is a clinical or policy rationale, leading to transparency in the design and inclusion of bioethics and domain knowledge.^42^ While HGLR is highly recommended for model development for critical scenarios, EBM is an excellent tool for exploratory research that is faster and comparable to resource intensive penalised regression methods. Although, we applied EBM after clustering and machine learning to address our goals, it is an easy to implement, independent tool that can be applied wherever parsimonious exploratory models are required.

### Limitations

We did not have access to non-geographic chiropractor characteristics or patient-reported outcomes such as pain severity. Therefore, we could not assess why patients visited certain providers more often or why they left a provider or treatment plan when they did. The early discontinuation pattern in cluster 3 may reflect either rapid resolution of symptoms after just 1-3 episodes of spinal CMT or worsening symptoms that justified pharmacologic or interventional treatment. We did not have access to data on Medicaid beneficiaries or uninsured individuals, so the results of this research may not be generalisable to those settings. However, our sample includes both commercially insured and Medicare Advantage enrollees, and offers geographic, racial, ethnic, and income diversity.

## Conclusion

Our study shows that the large patient population with NP/LBP has 3 distinct patterns of chiropractic visits representing the frequency and dose of chiropractic care. Patients in one cluster quickly decreased their utilisation of services over just 1-3 weeks, while those in another cluster received more services in a guideline-concordant manner. The most interesting finding was the small group in which patients received persistently more chiropractic care over a long period. The causes and outcomes of these 3 patterns are hard to ascertain from this study, which did not have patient reported outcomes, and will be addressed in future research. Our research shows how data scientists and clinical researchers can collaborate using advanced ML methods to discover new insights and generate testable hypotheses or consider new cohort designs.

## Supplementary Material

ooag035_Supplementary_Data

## Data Availability

We obtained data from the OptumLabs Data Warehouse (OLDW) which contains de-identified administrative claims data, including medical and pharmacy claims and eligibility information. Data requests can be sent to OPTUMLabs at rick.little@optum.com.
