# Supplementary material for: Spectral clustering identifies patterns of chiropractic care in a national longitudinal cohort
Source: JAMIA Open. 2026 May 14;9(3):ooag035. doi: 10.1093/jamiaopen/ooag035 (PMC13175172; doi:10.1093/jamiaopen/ooag035)
Supplement: ooag035_Supplementary_Data [file ooag035_supplementary_data.zip › Suppl File 1 Data Dictionary.docx]

| **Variable Name** | **Description** |
| --- | --- |
| Cohort | 1=NP cohort 2=LBP cohort 3=NP/LBP cohort |
| Gender | 1=Female, 0=Male |
| Insurance_COM | Insurance plan is Commercial insurance (COM) at the index date; 1=Yes, 0=No |
| Race | 1=Asian 2=Black 3=Hispanic 4=White 5=Unknown |
| Recurrent_patient | If a patient is recurrent in any following years; 1=Yes, 0=No |
| Age | Range between 18-89 |
| VisitChiro_Week1 | Number of Chiro visit in week 1 after index date (including index date, so value=1-7) |
| VisitChiro_Week2 | Number of Chiro visit in week 2 after index date |
| VisitChiro_Week3 | Number of Chiro visit in week 3 after index date |
| VisitChiro_Week4 | Number of Chiro visit in week 4 after index date |
| VisitChiro_Week5 | Number of Chiro visit in week 5 after index date |
| VisitChiro_Week6 | Number of Chiro visit in week 6 after index date |
| VisitChiro_Week7 | Number of Chiro visit in week 7 after index date |
| VisitChiro_Week8 | Number of Chiro visit in week 8 after index date |
| VisitChiro_Week9 | Number of Chiro visit in week 9 after index date |
| VisitChiro_Week10 | Number of Chiro visit in week 10 after index date |
| VisitChiro_Week11 | Number of Chiro visit in week 11 after index date |
| VisitChiro_Week12 | Number of Chiro visit in week 12 after index date |
| VisitChiro_Week13 | Number of Chiro visit in week 13 after index date |
| VisitChiro_Week14 | Number of Chiro visit in week 14 after index date |
| VisitChiro_Week15 | Number of Chiro visit in week 15 after index date |
| VisitChiro_Week16 | Number of Chiro visit in week 16 after index date |
| VisitChiro_Week17 | Number of Chiro visit in week 17 after index date |
| VisitChiro_Week18 | Number of Chiro visit in week 18 after index date |
| VisitChiro_Week19 | Number of Chiro visit in week 19 after index date |
| VisitChiro_Week20 | Number of Chiro visit in week 20 after index date |
| VisitChiro_Week21 | Number of Chiro visit in week 21 after index date |
| VisitChiro_Week22 | Number of Chiro visit in week 22 after index date |
| VisitChiro_Week23 | Number of Chiro visit in week 23 after index date |
| VisitChiro_Week24 | Number of Chiro visit in week 24 after index date |
| VisitChiro_Week25 | Number of Chiro visit in week 25 after index date |
| VisitChiro_Week26 | Number of Chiro visit in week 26 after index date |
| Spine_Comorb_Index | Patient has at least one visit with Dx of Myelopathy, Spinal Radiculopathy, Cauda Equina Syndrome, or Headache at index date; 1=Yes, 0=No |
| Spine_Radiculop_Index | Patient has at least one visit with any Dx of Spinal Radiculopathy at index date; 1=Yes, 0=No |
| Headache_Index | Patient has at least one visit with any Dx of Headache at index date; 1=Yes, 0=No |
| DislocSpineInj_Index | Patient has at least one visit with any Dx of Dislocation Spinal Injury at index date; 1=Yes, 0=No |
| Cnt_Previsits_PriCare | Number of visits to Entry point provider - Primary Care Physician - 12 months before Index date |
| Cnt_Previsits_EmrMed | Number of visits to Entry point provider - Emergency Medicine Physician - 12 months before Index date |
| Cnt_Previsits_Anesthe | Number of visits to Entry point provider - Anesthesiologist- 12 months before Index date |
| Cnt_Previsits_Chiro | Number of visits to Entry point provider - Chiropractor - 12 months before Index date |
| Cnt_Previsits_Neuro | Number of visits to Entry point provider - Neurologist - 12 months before Index date |
| Cnt_Previsits_NeuroSurg | Number of visits to Entry point provider - Neuro Surgeon- 12 months before Index date |
| Cnt_Previsits_Orthop | Number of visits to Entry point provider - Orthopedist - 12 months before Index date |
| Cnt_Previsits_OTPT | Number of visits to Entry point provider - Occupational/physical Therapist - 12 months before Index date |
| Cnt_Previsits_Rehab | Number of visits to Entry point provider - Rehabilitation Medicine Physician - 12 months before Index date |
| Cnt_Previsits_Rheumat | Number of visits to Entry point provider - Rheumatologist - 12 months before Index date |
| Cnt_Previsits_UrgentCare | Number of visits to Entry point provider - Urgent Care Physician- 12 months before Index date |
| Cerv_NP_CT0_30D | Computed temography (CT) with Cervical or Neck Pain within 30 days after index date (Including index date); 1=Yes, 0=No |
| NonCerv_LBP_CT0_30D | Computed temography (CT) with Non-Cervical or Low Back Pain within 30 days after index date (Including index date); 1=Yes, 0=No |
| Cerv_NP_XRay0_30D | Xray with Cervical or Neck Pain within 30 days after index date (Including index date); 1=Yes, 0=No |
| NonCerv_LBP_XRay0_30D | XRay with Non-Cervical or Low Back Pain within 30 days after index date (Including index date); 1=Yes, 0=No |
| Cerv_NP_MRI0_30D | Magnetic Resonance Imaging (MRI) with Cervical or Neck Pain within 30 days after index date (Including index date); 1=Yes, 0=No |
| NonCerv_LBP_MRI0_30D | Magnetic Resonance Imaging (MRI) with Non-Cervical or Low Back Pain within 30 days after index date (Including index date); 1=Yes, 0=No |
| Any_Opioid0_30 | Any opioid Rx within 0-30 days after index date among patients without any opioid Rx during 365 days befor index date Yes=1/No=0 |
| Any_MR0_30 | Any Muscle relaxants Rx within 0-30 days after index date among patients without any muscle relaxants Rx during 365 days befor index date Yes=1/No=0 |
| Any_Benzo0_30 | Any Benzodiazepines Rx within 0-30 days after index date among patients without any Benzodiazepines Rx during 365 days befor index date Yes=1/No=0 |
| Elix_Total | Total Elixhauser score for each patient |
| Elix_Physical | Physcial Elixhauser score=Total Elixhauser score-Alcohol-Depress-Drug Abuse-Psychoses |
| Elix_AIDS | Acquired immune deficiency syndrome 1=Yes, 0=No |
| Elix_ALCOHOL | Alcohol abuse 1=Yes, 0=No |
| Elix_ANEMDF | Deficiency anemias 1=Yes, 0=No |
| Elix_ARTH | Arthropathies 1=Yes, 0=No |
| Elix_BLDLOSS | Chronic blood loss anemia 1=Yes, 0=No |
| Elix_CANCER_LEUK | Leukemia 1=Yes, 0=No |
| Elix_CANCER_LYMPH | Lymphoma 1=Yes, 0=No |
| Elix_CANCER_METS | Metastatic cancer 1=Yes, 0=No |
| Elix_CANCER_NSITU | Solid tumor without metastasis, in situ 1=Yes, 0=No |
| Elix_CANCER_SOLID | Solid tumor without metastasis, malignant 1=Yes, 0=No |
| Elix_CBVD | Cerebrovascular disease 1=Yes, 0=No |
| Elix_CHF | Congestive heart failure 1=Yes, 0=No |
| Elix_COAG | Coagulopathy 1=Yes, 0=No |
| Elix_DEMENTIA | Dementia 1=Yes, 0=No |
| Elix_DEPRESS | Depression 1=Yes, 0=No |
| Elix_DIAB_CX | Diabetes with chronic complications 1=Yes, 0=No |
| Elix_DIAB_UNCX | Diabetes without chronic complications 1=Yes, 0=No |
| Elix_DRUG_ABUSE | Drug abuse 1=Yes, 0=No |
| Elix_HTN_CX | Hypertension, complicated 1=Yes, 0=No |
| Elix_HTN_UNCX | Hypertension, uncomplicated 1=Yes, 0=No |
| Elix_LIVER_MLD | Liver disease, mild 1=Yes, 0=No |
| Elix_LIVER_SEV | Liver disease, moderate to severe 1=Yes, 0=No |
| Elix_LUNG_CHRONIC | Chronic pulmonary disease 1=Yes, 0=No |
| Elix_NEURO_MOVT | Neurological disorders affecting movement |
| Elix_NEURO_OTH | Other neurological disorders 1=Yes, 0=No |
| Elix_NEURO_SEIZ | Seizures and epilepsy 1=Yes, 0=No |
| Elix_OBESE | Obesity 1=Yes, 0=No |
| Elix_PARALYSIS | Paralysis 1=Yes, 0=No |
| Elix_PERIVASC | Peripheral vascular disease 1=Yes, 0=No |
| Elix_PSYCHOSES | Psychoses 1=Yes, 0=No |
| Elix_PULMCIRC | Pulmonary circulation disease 1=Yes, 0=No |
| Elix_RENLFL_MOD | Renal failure, moderate 1=Yes, 0=No |
| Elix_RENLFL_SEV | Renal failure, severe 1=Yes, 0=No |
| Elix_THYROID_HYPO | Hypothyroidism 1=Yes, 0=No |
| Elix_THYROID_OTH | Other thyroid disorders 1=Yes, 0=No |
| Elix_ULCER_PEPTIC | Peptic ulcer with bleeding 1=Yes, 0=No |
| Elix_VALVE | Valvular disease 1=Yes, 0=No |
| Elix_WGHTLOSS | Weight loss 1=Yes, 0=No |
